# Energy Flux in the Cochlea: Evidence Against Power Amplification of the Traveling Wave

**DOI:** 10.1007/s10162-015-0529-5

**Published:** 2015-07-07

**Authors:** Marcel van der Heijden, Corstiaen P. C. Versteegh

**Affiliations:** Department of Neuroscience, Erasmus MC, Room Ee 1285, P.O. Box 2040, 3000 CA Rotterdam, The Netherlands

**Keywords:** cochlear mechanics, laser vibrometry, basilar membrane, traveling wave, cochlear amplifier, group velocity

## Abstract

Traveling waves in the inner ear exhibit an amplitude peak that shifts with frequency. The peaking is commonly believed to rely on motile processes that amplify the wave by inserting energy. We recorded the vibrations at adjacent positions on the basilar membrane in sensitive gerbil cochleae and tested the putative power amplification in two ways. First, we determined the energy flux of the traveling wave at its peak and compared it to the acoustic power entering the ear, thereby obtaining the net cochlear power gain. For soft sounds, the energy flux at the peak was 1 ± 0.6 dB less than the middle ear input power. For more intense sounds, increasingly smaller fractions of the acoustic power actually reached the peak region. Thus, we found no net power amplification of soft sounds and a strong net attenuation of intense sounds. Second, we analyzed local wave propagation on the basilar membrane. We found that the waves slowed down abruptly when approaching their peak, causing an energy densification that quantitatively matched the amplitude peaking, similar to the growth of sea waves approaching the beach. Thus, we found no local power amplification of soft sounds and strong local attenuation of intense sounds. The most parsimonious interpretation of these findings is that cochlear sensitivity is not realized by amplifying acoustic energy, but by spatially focusing it, and that dynamic compression is realized by adjusting the amount of dissipation to sound intensity.

## INTRODUCTION

The cochlea is both a transducer that converts sound to neural activity and a frequency analyzer that separates acoustic components. Its elongated fluid-filled cavities are separated by a thin elastic structure, the basilar membrane (BM), whose motion is coupled to sensory cells. The BM supports traveling waves that have two crucial, but poorly understood properties. First, their amplitude changes drastically during propagation, exhibiting a peak that shifts position with frequency. This frequency mapping underlies the spectral analysis. Second, soft sounds evoke sharper peaking than do intense sounds. This intensity dependency reflects the ear’s dynamic range compression.

In the 1980s, the failure of classical fluid-mechanical models to account for these unusual wave properties led to the introduction of “active models,” in which the peaking of the wave is associated with a region of negative damping (Kim et al. 1980b; Neely 1985). In this scenario, motile processes in outer hair cells (OHCs) amplify the waves. The injection of mechanical energy by this “cochlear amplifier” is assumed to improve the sensitivity to soft sounds, and its saturation is invoked to explain compression (Ashmore et al. 2010).

Cochlear amplification has slowly gained acceptance and is now the dominant view. There is a recent trend to present cochlear amplification as proven (e.g., Hudspeth 2013) even though the evidence quoted in favor of it comes from older studies: the loss of cochlear sensitivity following OHC damage (Evans and Harrison 1976) and the existence of spontaneous emissions (Kemp, 1979). Until recently, that same evidence was more cautiously described as impressive, but inconclusive (Robles and Ruggero 2001; Ashmore 2008; Shera 2007), since both physiological vulnerability and spontaneous emissions leave room for alternative explanations not based on amplification. For instance, while it is undisputed that OHCs *control* BM motion, that is not to say that they also *drive* the vibrations in the sense of supplying the mechanical energy. In an alternative scenario, OHCs control BM motion by functioning as brakes that cause mechanical energy to be absorbed rather than injected (Allen 1979). A combination of the two roles, i.e., amplification at low intensities and variable attenuation at high intensities, is also conceivable. Spontaneous emissions certainly seem to reveal processes capable of producing mechanical energy (Talmadge et al. 1991), and there have been many modeling attempts to link them to some form of amplification (Shera 2003a; Duke and Jülicher 2008), but to conclude from their mere existence that the cochlea systematically amplifies its acoustic input at all frequencies is rather tentative. Spontaneous emissions may also be side effects of other forms of mechanical control exerted by OHCs, for instance, negative feedback of an automatic brake system. Particularly in the presence of delayed coupling and tuned circuits, feedback, even when negative, easily gives rise to ringing and other instabilities (Doyle et al. 1992). It is also noteworthy that spontaneous emissions are extremely rare in normal-hearing nonprimate laboratory animals (Martin et al. 1988) and that even in normal-hearing humans, their incidence is ∼40 % (Wier et al. 1984).

Arguably, the most problematic aspect of the putative amplifier (and a good reason to keep an open mind toward alternatives) has been its physiological implementation (Ashmore et al. 2010). Amplification requires phase-locked motile feedback at high frequencies (>150 kHz in some species). While somatic OHC motility by itself may be fast enough (Frank et al. 1999), it is difficult to see how the AC component of the OHC receptor potential evoked by near-threshold sounds can have sufficient amplitude to drive motility at such high frequencies, as it is shunted by the membrane capacitance (Cody and Russell 1987). It is unknown whether the alternative mechanism, hair bundle motility (Kennedy et al. 2006), can operate at these very high frequencies. For a parametric (rather than cycle-by-cycle) operation of OHCs, such as braking or adjusting the radial profile of BM motion (Ren and Gillespie 2007), high-frequency limitations are not a problem.

Rather than relying on circumstantial evidence, a number of studies have attempted to estimate the amount of amplification based on measurements in the auditory periphery. Combining auditory nerve recordings and otoacoustic emissions, Allen and Fahey (1992) found a negative result, although their conclusions have been disputed by others (Shera 2003b; de Boer et al. 2005). Cochlear mechanical measurements are an obvious choice for tests of amplification. In sensitive cochleae, the range of sensitivity, assessed by normalizing BM vibrations to the middle ear response, exceeds 50 dB, and compressive growth persists to at least 100 dB sound pressure level (SPL) (Rhode 2007). Thus, if a simple “saturating amplifier” were the only explanation of cochlear compression, one would expect it to provide at least 50 dB of amplification at low intensities (as is indeed the case for simplified positive-feedback models like that of Cooper 1998) and persist to amplify to very high intensities. Estimates of power gain at low intensities derived from BM and neural data are variable: 40 dB (Brass and Kemp 1993), 12 dB on average with confidence intervals spanning [−4, ∞] dB (Shera 2007), and 0.4–17.7 dB (de Boer and Nuttall 2001). The intensity range over which BM recordings and intracochlear pressure measurements appear to indicate amplification, is restricted to low and moderate intensities in some studies (e.g., Olson 2001), whereas in other studies, it appears to extend to high intensities (de Boer and Nuttall 2000; Dong and Olson 2009). The large variability among these studies is not well understood but suggests that further work is needed and that novel methods are welcome.

Active cochlear models implement amplification by introducing a negative real part of cochlear impedance (negative damping) over a limited, frequency-dependent cochlear region just basal to the peak of the wave. When traversing this region, the wave picks up energy. In this region, then, there should be a local power gain, i.e., a positive gradient in energy flux. Here, we tested this prediction by determining the energy flux of the traveling wave and by comparing this flux both to the power input to the middle ear (net gain) and across adjacent locations on the BM (local gain). We base our analyses on BM recordings at two adjacent locations, which allow a more direct analysis of the energy transport under scrutiny than previous analyses based on single-point BM recordings or neural data.

## METHODS

### BM Recordings

**FIG. 1:**
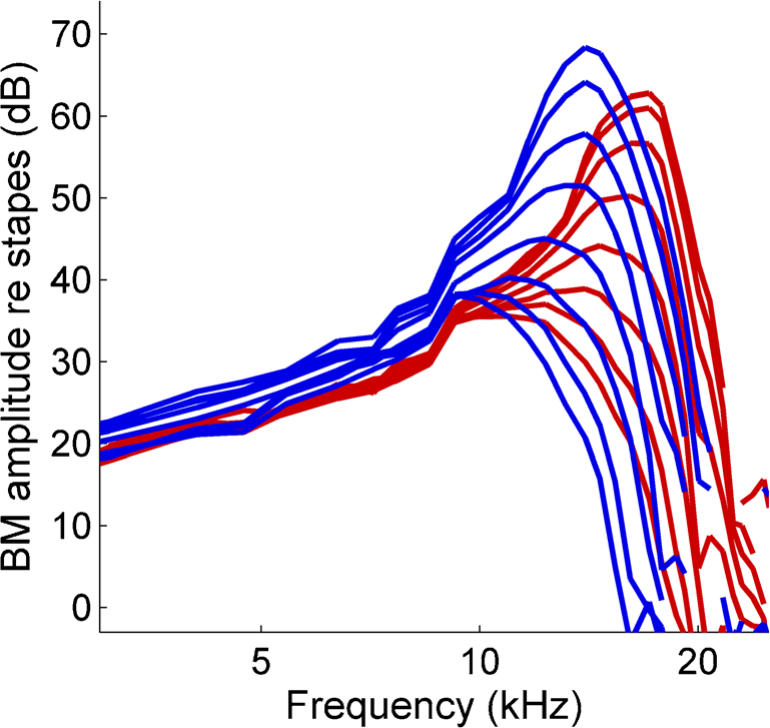
BM responses showing high sensitivity and compressive nonlinearity at low intensities. BM responses from two adjacent locations, normalized to stapes response. Nonlinear compressive growth of the cochlear response is apparent from the systematic decrease of stapes-to-BM gain with sound intensity. Any cochlear trauma causes a linearization at lower levels, which would cause the lower-intensity curves to overlap. Conversely, the persistence of nonlinearity down to the lowest intensities is a stringent test of cochlear sensitivity. Our criterion for data inclusion was the persistence of compression down to 10-dB SPL per component or better. Data shown were obtained from one of the five cochleae used for group velocity measurements (Fig. 5). Intensity was varied from 0- to 70-dB SPL per component in 10-dB steps. At both locations, the amplitude curves showed compression down to 0-dB SPL, the lowest intensity used (animal RG12433).

Details of the animal preparation, experimental setup, and stimuli are described in a recent publication (Versteegh and Van der Heijden 2013). BM motion was measured from pairs of locations spaced 145–252 μm in seven cochleae of Mongolian gerbil (*Meriones unguiculatus*; ∼60 g) in the 12–21-kHz region, using a Doppler laser interferometer. All procedures were approved by the Erasmus MC laboratory animal committee. Animals were anesthetized, and the pinna was removed, followed by opening the bulla, which gave access to the round window. After tearing the round window membrane, reflective beads were inserted into the cochlea and allowed to settle on the BM. A glass cover slip placed over the round window stabilized the air-fluid interface. The use of reflective beads allowed the recording of phase-locked, sub-nanometer BM vibrations in response to sounds of very low intensities (down to 0 dB SPL) by improving the signal to noise ratio and preventing the interference of spurious reflections from neighboring structures. The specific mass of the beads (1.03 times that of water), their small size (20–25 μm, i.e., an order of magnitude smaller than the wavelength of the traveling wave), and their incompressibility minimize their interference with the traveling wave and BM motion; indeed, these beads were shown to have little effect on BM motion in sensitive cochleae (Cooper 1999). For data to be included in the analysis, bead position had to be stable during data collection (variations <10 μm, monitored using the online camera built into the vibrometer and from the adjustment of the micrometers for horizontal beam positioning). The physiological condition of the cochlea was judged from the lower intensity limit at which the BM response showed compressive nonlinearity (Rhode 2007). Only data were accepted from sensitive cochleae that showed compressive nonlinearity down to 10-dB SPL per component or lower (Fig. 1).

In order to be accepted for analysis, spectral components from the recordings must show Rayleigh significant (*p* < 0.001) phase locking to the stimulus (Versteegh and Van der Heijden 2012). These acceptance criteria exclude any data from insensitive and damaged cochleae, from which a significant phase locked responses to 10-dB-SPL stimuli—whether linear or not—cannot be recorded at all. All data were collected within 2.5 h from the tearing of the round window membrane. Best frequency (BF) was determined from the peak of the velocity-frequency curves normalized to stapes motion.

Custom MATLAB software computed stimuli that were sent to a TDT System 3 (24-bit D/A channel at 111.6 kHz; Tucker-Davis Technologies, Alachua, FL, USA). A probe sealed with Vaseline to the bony rim of the ear canal delivered sound stimuli. After correction for the acoustical transfer of the probe, the spectrum varied <4 dB in the 5–25-kHz range. A single-point laser vibrometer (OFV-534; Polytec, Waldbronn, Germany) connected to a velocity decoder (VD-06; Polytec) and TDT System 3 (24-bit A/D channel at 111.6 kHz) measured BM velocity of two locations in response to the same stimuli consecutively.

For the data underlying the group velocity measurements (Fig. 3), stimuli were tone complexes with an average frequency spacing of either 700 or 300 Hz and a bandwidth of ∼2500 Hz, presented at a total intensity of 16–86 dB SPL in 10-dB steps. Irregular spacing of frequency components ensured that combination tones up to the third order did not coincide with any of the primary components (Meenderink and Van der Heijden 2011). A single stimulus lasted 60 s but could be repeated up to three times and the responses averaged. Group delays were determined using two methods. In the temporal method, the envelopes were extracted from the BM velocity response using a Hilbert transform and squared to lead to instantaneous power (up to an irrelevant scaling factor). Cross-correlation functions of the squared envelopes between the recording locations were computed, and the location of the maximum was determined. The fluctuations recorded at all three locations (stapes and the two beads) were highly similar (Fig. 3B; correlations between squared envelope >0.9). Thus, the power fluctuations of the stimulus were not deformed by excessive dispersion or nonlinearity, allowing the straightforward assessment of energy travel time from the response envelopes. In the spectral method, phase difference between the locations was plotted against frequency, and a third-order polynomial was fitted to these curves. The slope of these fits was then evaluated at BF. The two methods are compared in Figure 3D. As expected from the physical significance of group velocity as the speed of energy transport (Whitham 1974), the two methods were equivalent. The average data in Figure 3F were linearly extrapolated from 16–86 to 10–90 dB SPL in order to be applied to the energy flux analysis shown in Figure 4.

The local transfer functions (see “Results” section) shown in Figures 6 and 7 were obtained using the same type of irregularly spaced tone complexes, this time spanning a larger frequency range. In all recordings, the lowest-intensity stimuli were presented first, and the responses at both locations were recorded before moving to the next (higher) stimulus level. This order of stimulus presentation prevents any temporary reduction of cochlear sensitivity induced by prolonged high-intensity stimulation (Versteegh and Van der Heijden 2013) from affecting lower-intensity recordings.

### Analysis of Fluid Motion

This section describes the details of the estimates of kinetic energy density leading to the findings presented in “Results” section (Fig. 4A–C). Estimating kinetic energy required analyzing the motion of the fluid surrounding the BM. Large parts of the BM are surrounded by free fluid or by supporting cells which, lacking any structurally stiff parts, behave like malleable bags of fluid (Steele and Taber 1979a). Supporting cells that have structural stiffness are organized in tunnels that do not impede longitudinal flow and/or in structures with abundant gaps, allowing the fluid to flow around them. Motivated by this anatomy (Lim 1986), the fluid was treated as freely moving. This reduces the analysis to solving the Laplace equation for irrotational fluid motion, using the two-dimensional pattern of BM reported by Ren et al. (2011a) as boundary conditions.

**FIG. 2:**
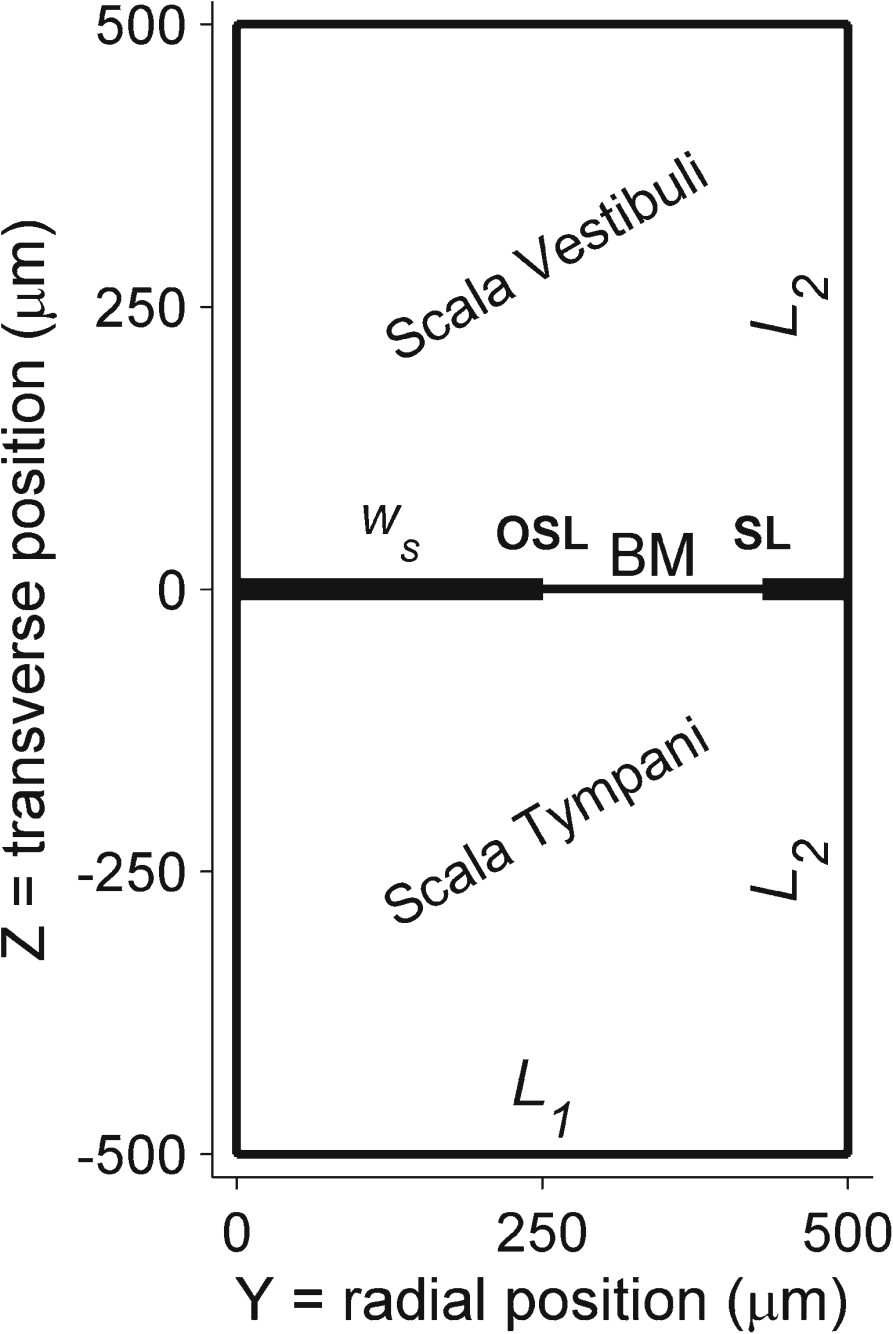
Schematic cross section of cochlear ducts used for calculating the fluid motion near the BM. This cross section illustrates the boundary conditions for the Laplace equation describing the irrotational fluid motion. All boundaries are rigid except the basilar membrane (*BM*), whose motion is prescribed by the two-dimensional data of Ren et al. (2011a). The BM is suspended between the osseous spiral lamina (OSL) and the spiral ligament (SL). Dimensions *L*_1_, *L*_2_, and *w*_s_ are provided in the text.

The analysis employs the analytical framework described by Steele and Taber (1979a) for computing three-dimensional fluid motion from the transverse motion profile of the BM and the geometry of the rigid boundaries. Fluid motion is described by a velocity potential obeying Laplace’s equation ∇^2^*ϕ* = 0. Rectangular boxes represent the scalae; (*x*,*y*,*z*) denote longitudinal, radial, transverse directions (Fig. 2). Width and height, *L*_1_ = *L*_2_ = 500 μm, were chosen to match the cross-sectional area in the gerbil’s basal turn; the inner bony shelf width *w*_S_ = 250 μm (Plassmann et al. 1987).

The boundary conditions are

1 $$ \begin{array}{l}\frac{\partial \phi }{\partial y}=0\kern0.5em \mathrm{f}\mathrm{o}\mathrm{r}\kern0.5em y=0,{L}_1;\\ {}\frac{\partial \phi }{\partial z}={v}_{\mathrm{BM}}\eta (y) \cos \left(kx-\omega t\right)\kern0.5em \mathrm{f}\mathrm{o}\mathrm{r}\kern0.5em z=0;\kern1em \frac{\partial \phi }{\partial z}=0\kern0.5em \mathrm{f}\mathrm{o}\mathrm{r}\kern0.5em z=\pm {L}_2.\end{array} $$

Here, *v*_BM_ is the amplitude of transverse BM velocity at its radial maximum; *η*(*y*) combines the normalized radial profile of BM displacement with the vanishing displacement of the bony shelves. The cosine represents the longitudinal traveling wave having wave number *k* and angular frequency *ω*. Although this analysis is strictly valid only for a homogeneous duct with nonvarying properties, its application to the cochlea with its stiffness gradient and subsequent longitudinal wavelength variation is justified by the gradual nature of these gradients. As explained in Sec. III of Steele and Taber, these are the same conditions that justify the WKB approximation. Equation 7 of Steele and Taber provides the solution *ϕ* as a series of elementary functions, and we used this expression to compute the spatial distribution of kinetic energy shown in Figure 4A. Whereas Steele and Taber used standard modes of an elastic beam for the radial profile *η*(*y*), we inserted the radial profile of BM motion measured by Ren et al. (2011a) and values of wave number *k* derived from these same data. For the time average kinetic energy per unit length *E*_K_, Eq. 16b of Steele and Taber provides the expression

2 $$ {E}_{\mathrm{K}}={\scriptscriptstyle \frac{1}{2}}\rho {\mathrm{h}}_{eq}{v}_{\mathrm{BM}}^2{\displaystyle \underset{0}{\overset{L_1}{\int }}{\eta}^2(y) dy}, $$

with *ρ* the fluid mass density and *h*_eq_ an equivalent thickness of fluid moving with the BM. *h*_eq_ depends on *η*(*y*) and on the wavelength *λ* = 2π/*k* (Eq. 14b of Steele and Taber 1979a). The normalized radial profile *η*(*y*) was obtained from Figure 2C of Ren et al. (2011a), yielding ${\displaystyle \underset{0}{\overset{L_1}{\int }}{\eta}^2(y) dy}$ = 35 μm, which may be viewed as an effective width of the fluid motion. The wavelength *λ* was obtained by fitting parabolas to the phase curves in Figure 4B of Ren et al. (2011a) and evaluating their slopes at the 16-kHz location at 2500 μm from the stapes. With increasing intensity (10–90 dB SPL), *λ* increased from 254 to 403 μm, causing *h*_eq_ to vary from 31 to 43 μm. BM displacement amplitudes *ξ*_BM_ were taken from the raw data underlying Figure 4A of Ren et al. (2011a), evaluated over a 20-μm stretch around the 2500-μm location. Displacement was converted to velocity using *v*_BM_ = *ω ξ*_BM_. The confined character of the spatial distribution of *E*_K_ (Fig. 4A) and the fact that *kL*_1_ > > 1, *kL*_2_ > > 1 imply that the scalae do not constrain the fluid motion; the wave is a deep-water wave and genuinely three-dimensional (Steele and Taber 1979b). Indeed, varying *L*_1_ and *L*_2_ by ±50 % and *w*_S_ from 50 to 400 μm affected *E*_K_ by less than 0.2 dB. Thus, the exact geometry of the fixed boundaries (Fig. 2) is not critical to the estimate.

### Relation Between Wave Amplitude and Group Velocity

This section presents the mathematical underpinning of the analysis of group velocity gradient and local amplitude gain presented in “Results” section when discussing the low-intensity curves of Figure 6. We assume that the power flux *P* is constant (i.e., the wave is neither amplified nor attenuated) and analyze the consequences of this Ansatz. *P* is the product of group velocity and energy density; from the equality of time average kinetic and potential energy, given a local displacement amplitude *A* and local stiffness *s*,

3 $$ P=\frac{1}{2}Us{A}^2. $$

When the power flux *P* is constant (neither amplified nor attenuated), the amplitude ratio *G*_12_ between adjacent locations 1 and 2 obeys

4 $$ {G}_{12}^2={\left({A}_2/{A}_1\right)}^2=\frac{s_1{U}_1}{s_2{U}_2}. $$

Here, 1 and 2 are the basal and apical locations, respectively. In the interpretation of data as shown in Figures 6 and 7, it is important to realize that the amplitudes measured at two adjacent locations may differ by an additional unknown factor due to slight differences in radial position of the two reflective beads. This factor is independent of frequency and intensity (Cooper 2000). From the measurements, therefore, *G*_12_ can be determined up to an unknown constant factor.

In the familiar example of sea waves entering a shallow beach, gravity plays the role of restoring force (“stiffness”), and its constancy, *s*_1_ = *s*_2_, reduces the RHS of Eq. 4 to the ratio of group velocities. In that case, the spatial variation of group velocity is entirely caused by the dependence of effective fluid mass on water depth. In the cochlea, *s*_1_/*s*_2_ differs from unity owing to the longitudinal stiffness gradient. This is taken into account as follows. In the low-frequency limit, the waves are long and one-dimensional. There is no dispersion: at a fixed location, group velocity *U*_LF_ and phase velocity *c*_LF_ are equal and constant in the low-frequency limit:

5 $$ {U}_{\mathrm{LF}}={c}_{\mathrm{LF}}=\sqrt{s/m}, $$

where *m* is the cross-sectional fluid mass per length unit, and the subscript LF denotes the low-frequency limit. Using Eq. 4, the local amplitude gain *G*_12,LF_ in the low-frequency limit becomes

6 $$ {G}_{12,\mathrm{L}\mathrm{F}}^2={\left(\frac{s_1}{s_2}\right)}^{3/2}. $$

This expression is independent of frequency and wavelength; it corresponds to the flat, linear, low-frequency portion of the local gain functions (Figs. 6 and 7; Figs. 2 and 3 of Ren et al. 2011b). When normalizing *G*_12_ by *G*_12,LF_ (which also eliminates the unknown geometric factor arising from possible differences in radial position of the two beads), one obtains

7 $$ \begin{array}{cc}{\left({G}_{12}/{G}_{12,\mathrm{L}\mathrm{F}}\right)}^2& =\sqrt{s_2/{s}_1}\frac{U_1}{U_2}\\ {}& \equiv {\gamma}_{12}\frac{U_1}{U_2}.\end{array} $$

Apart from the factor *γ*_12_, the normalization of the gain reduces the expression for the amplitude gain to the ratio of local group velocities, just like the case of sea waves approaching the beach. For nearby recording locations, *γ*_12_ is slightly smaller than one. Based on a frequency map created by longitudinal stiffness variations proportional to the square of BF (Emadi et al. 2004), *γ*_12_ equals the ratio of the BFs at the two locations

8 $$ {\gamma}_{12}=\frac{f_{\mathrm{best},2}}{f_{\mathrm{best},1}}, $$

which gives *γ*_12_ = 0.82 and *γ*_12_ = 0.79 for the data shown in Figures 6 and 7, respectively.

When relating the phase and gain curves of the local transfer functions in Figure 6, it is important to realize that the asymptotic slopes of the phase curves *τ*_LF_ and *τ*_HF_ do not directly correspond to *U*_1_ and *U*_2_: the latter are the group velocities at the two locations at a given frequency; the former correspond to group velocity in two frequency ranges at a given location. The two pairs are related by tonotopy (“scaling”). On account of the scaling invariance of *U*/*ω* (see Eq. 15 below), their ratios are related by

9 $$ \begin{array}{cc}\frac{U_1}{U_2}& =\frac{f_{\mathrm{best},1}}{f_{\mathrm{best},2}}\frac{\tau_{\mathrm{HF}}}{\tau_{\mathrm{LF}}}\\ {}& =\frac{1}{\gamma_{12}}\frac{\tau_{\mathrm{HF}}}{\tau_{\mathrm{LF}}},\end{array} $$

leading to the simple relation between normalized peak gain and asymptotic slopes

10 $$ {\left({G}_{12}/{G}_{12,\mathrm{L}\mathrm{F}}\right)}^2=\frac{\tau_{\mathrm{HF}}}{\tau_{\mathrm{LF}}}. $$

Equation 10 is the mathematical underpinning of the scaling argument used in the discussion of Figure 6 in the main text.

### Degree of Damping of Waves

This section clarifies the derivation of the degree of damping presented in “Results” section when discussing the high-intensity curves in Figure 6. A vibrating system is called *critically damped* when its amplitude decreases by a factor *e*^2π^ during each cycle of its (unforced) oscillation, so the rate of decay of the energy equals 20 log(*e*^2π^) = 54.6 dB per cycle. In an underdamped (overdamped) system, the losses are smaller (larger). The degree of damping is expressed by the dimensionless damping coefficient *ζ* = *L*/54.6, where *L* is the temporal decay rate of energy decay of the system in decibel per cycle. Critical damping corresponds to *ζ* = 1. These definitions apply to traveling waves without modification. Now, the energy, in addition to being dissipated, is moving at the group velocity (Lighthill 1978). In a nondispersive wave, group velocity equals phase velocity, and the loss per spatial cycle equals the loss per temporal cycle. In a dispersive wave, the two are related through the ratio *χ* = *c*/*U* of phase velocity *c* to group velocity *U*, yielding a spatial decay rate of 54.6*χ* dB/cycle for a critically damped wave. Using the definitions of phase and group velocity in terms of phase, frequency, and distance (Whitham 1974), the ratio *χ* can be readily extracted from local phase transfer functions (*φ*_12_ versus *f*) using *χ* = *U/c* = *φ*_12_ / (*f* ∂*φ*_12_/∂*f*), where *f* is the frequency, *φ*_12_ is the phase difference between the locations, and ∂*φ*_12_/∂*f* is the slope of the phase-frequency curve. For the 80-dB-SPL curve at 14 kHz (Fig. 6D), one obtains *χ* = 3.0. Thus, for these waves, critical damping would correspond to a spatial power decay rate of 164 dB/cycle. The observed rate of 36 dB/cycle (see “Results” section) shows that the wave is underdamped, having a dimensionless damping coefficient of *ζ* = 36/164 = 0.22.

### Relation Between Local Amplitude Gain and Local Phase Difference

This section describes the derivation of the predictions of the phase curves presented in “Results” section (Fig. 7B). Scaling invariance (Zweig 1976) states a trade-off between the frequency dependence and place dependence of the phase *φ* of BM displacement, which equals the phase of the excess pressure when damping is small (Lighthill 1978), of the form

11 $$ \varphi \left(x,\omega \right)=-\varPhi \left(\alpha x+\nu \right), $$

where *α* is the gradient of the frequency-place map, and

12 $$ \nu = \log \omega /{\omega}_{\mathrm{ref}}, $$

with *ω*_ref_ an arbitrary reference frequency. The local wave number (spatial frequency) *k* then becomes

13 $$ k=\partial \varPhi /\partial x=\alpha \varPhi^{\prime}\left(\alpha x+\nu \right), $$

where the prime denotes the derivative. The group velocity *U* equals

14 $$ U=\partial \omega /\partial k, $$

hence

15 $$ {U}^{-1}=\partial k/\partial \omega =\alpha \varPhi^{{\prime\prime}}\left(\alpha x+\nu \right)\partial \nu /\partial \omega =\frac{\alpha }{\omega}\varPhi^{{\prime\prime}}\left(\alpha x+\nu \right). $$

Combining Eqs. 7 and 15 and defining

16 $$ {\tilde{G}}_{12}=\frac{G_{12}}{\sqrt{\gamma_{12}}{G}_{12,\mathrm{L}\mathrm{F}}}, $$

it follows

17 $$ {\tilde{G}}_{{}_{12}}^2\left(\nu \right)=U\left({x}_1,\nu \right)/U\left({x}_2,\nu \right)=\varPhi^{{\prime\prime}}\left(\alpha {x}_2+\nu \right)/\varPhi^{{\prime\prime}}\left(\alpha {x}_1+\nu \right). $$

Defining

18 $$ h\left(\nu \right)={\scriptscriptstyle \frac{1}{2}} \log \varPhi^{{\prime\prime}}\left(\nu +\alpha {x}_1\right), $$

it follows

19 $$ \log {\tilde{G}}_{12}\left(\nu \right)=h\left(\nu +\alpha D\right)-h\left(\nu \right), $$

where *D* is the distance *x*_2_-*x*_1_ between the recording locations. The phase difference *Φ*_12_ between locations *x*_1_ and *x*_2_ equals

20 $$ \begin{array}{c}{\varPhi}_{12}\left(\nu \right)=\varPhi \left(\alpha {x}_2+\nu \right)-\varPhi \left(\alpha {x}_1+\nu \right)\\ {}=\alpha {\displaystyle \underset{x_1}{\overset{x_2}{\int }}dx\varPhi^{\prime}\left(\alpha x+\nu \right)}\\ {}=\alpha {\displaystyle \underset{\nu_0}{\overset{\nu }{\int }}d\mu }{\displaystyle \underset{x_1}{\overset{x_2}{\int }}dx\varPhi^{{\prime\prime}}\left(\alpha x+\mu \right)},\end{array} $$

where the lower bound *ν*_0_ = log(*ω*_0_/*ω*_ref_) of the first integral is indefinite, leading to an arbitrary integration constant. Combining Eqs. 18 and 20 yields

21 $$ {\varPhi}_{12}\left(\omega \right)=\alpha {\displaystyle \underset{ \log {\omega}_0}{\overset{ \log \omega }{\int }}d\mu }{\displaystyle \underset{0}{\overset{D}{\int }}dx}{\mathrm{e}}^{2h\left(\alpha x+\mu \right)}. $$

The local transfer data (Figs. 6 and 7) consist of the phase difference *Φ*_12_ and amplitude gain *G*_12_, both as a function of frequency. Equations 19 and 21 link these two measurable quantities in terms of the auxiliary function *h*(*ν*). In order to predict *Φ*_12_ from the *G*_12_ data, we first obtained *h*(*ν*) by numerically solving Eq. 19. This yielded *h*(*ν*) up to addition of an arbitrary periodic function with period *αD*. This ambiguity was resolved by selecting the most regular *h*(*ν*) obeying Eq. 19, i.e., by minimizing

22 $$ {\displaystyle \int d\nu }{\left(h{\prime\prime} \left(\nu \right)\right)}^2 $$

over the range of *ν* values dictated by the data. This fixes *h*(*ν*) up to an additive constant (in the numerical computations, solving Eq. 19 and minimizing Eq. 22 were realized by interpolating the data on a fine grid and solving the discrete versions of these equations using linear algebra). The *h*(*ν*) thus obtained was inserted into Eq. 21 to yield the predicted local phase transfer *Φ*_12_(*ω*). The undetermined integration constant of Eq. 21 and the arbitrary additive constant to *h*(*ν*) result in an indefinite offset and scaling factor, respectively. Their values were chosen to best fit the phase data in a least square sense.

## RESULTS

### Net Power Gain

All traveling waves transport energy. The energy flux is the product of the energy density and the speed at which it travels. In dispersive media like the cochlea, the speed of the energy differs from the visible speed of wave crests. Instead, it equals the propagation speed of entire wave packets (Lighthill 1978), hence its name “group velocity.”

**FIG. 3:**
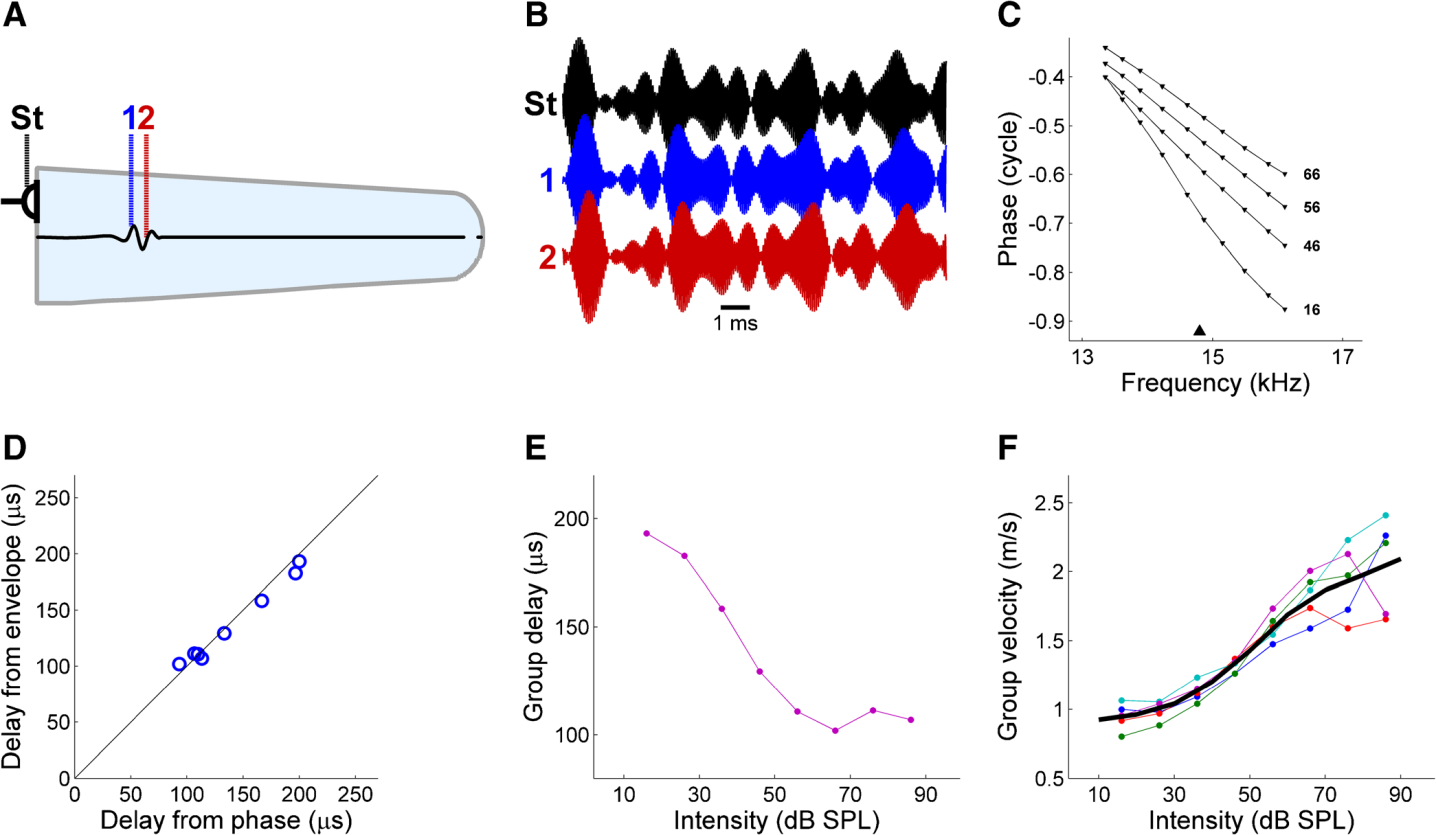
Measuring the speed of cochlear energy transport. **A** Schematized cochlea with recording locations indicated: stapes (*St*) and two adjacent basilar membrane (BM) locations (*1*, *2*). **B** Waveforms recorded at these locations evoked by narrowband sounds. Relative delays of magnitude fluctuations reflect energy travel times. **C** Phase difference between locations 1 and 2 versus frequency at various intensities (indicated in dB SPL). *Triangle* marks best frequency (BF). **D** Comparison of group delays estimated from phase-frequency plots (abscissa) and from temporal comparison of magnitude fluctuations (ordinate). Unity line is shown for reference (*black line*). **E** Intensity-dependent group delay between locations 1 and 2. **F** Group velocity at BF from five cochleae, BFs 12.7–18.2 kHz. *Thick black line*: mean, linearly extrapolated to the 10–90 dB-SPL range (**B**–**E**: animal RG12448).

We determined group velocity in sensitive cochleae by measuring vibrations of the stapes and two neighboring BM locations (Fig. 3A) in response to narrowband sound stimuli. These stimuli are an ongoing series of wave packets because of their magnitude fluctuations. The same fluctuations were found in the recorded waveforms. In order to quantify the power fluctuations, we extracted the Hilbert envelope from the recordings at all three locations. The squared envelopes were highly similar across the three locations (Fig. 3B; correlations >0.9). Thus, the power fluctuations of the stimulus were not deformed by excessive dispersion or nonlinearity, allowing the straightforward assessment of energy travel time from the response envelopes. The alternative method employs the slopes of phase-frequency curves (Fig. 3C). The two methods produced equivalent results (Fig. 3D), as expected from the generality of the group velocity concept and its physical significance as the velocity of energy transport in traveling waves under a wide range of conditions including damping and nonlinearity (Lighthill 1965; Whitham 1974). Group delay depended strongly on sound intensity (Fig. 3E). Group velocity in five sensitive cochleae increased with intensity, varying from 0.9 m/s at 10 dB SPL to 2.1 m/s at 90 dB SPL (Fig. 3F). Thus, the acoustic energy, which approaches the ear at 340 m/s, is decelerated to a mere walking pace prior to sensory detection. Comparable low-intensity group velocities of ∼1 m/s can be inferred from published data of sensitive cochleae (Ren et al. 2011a; Rhode and Recio 2000). Our high-intensity values are somewhat lower than the rough 3-m/s estimate by Lighthill (1981) based on 85-dB-SPL data from a damaged cochlea (Rhode 1978).

**FIG. 4:**
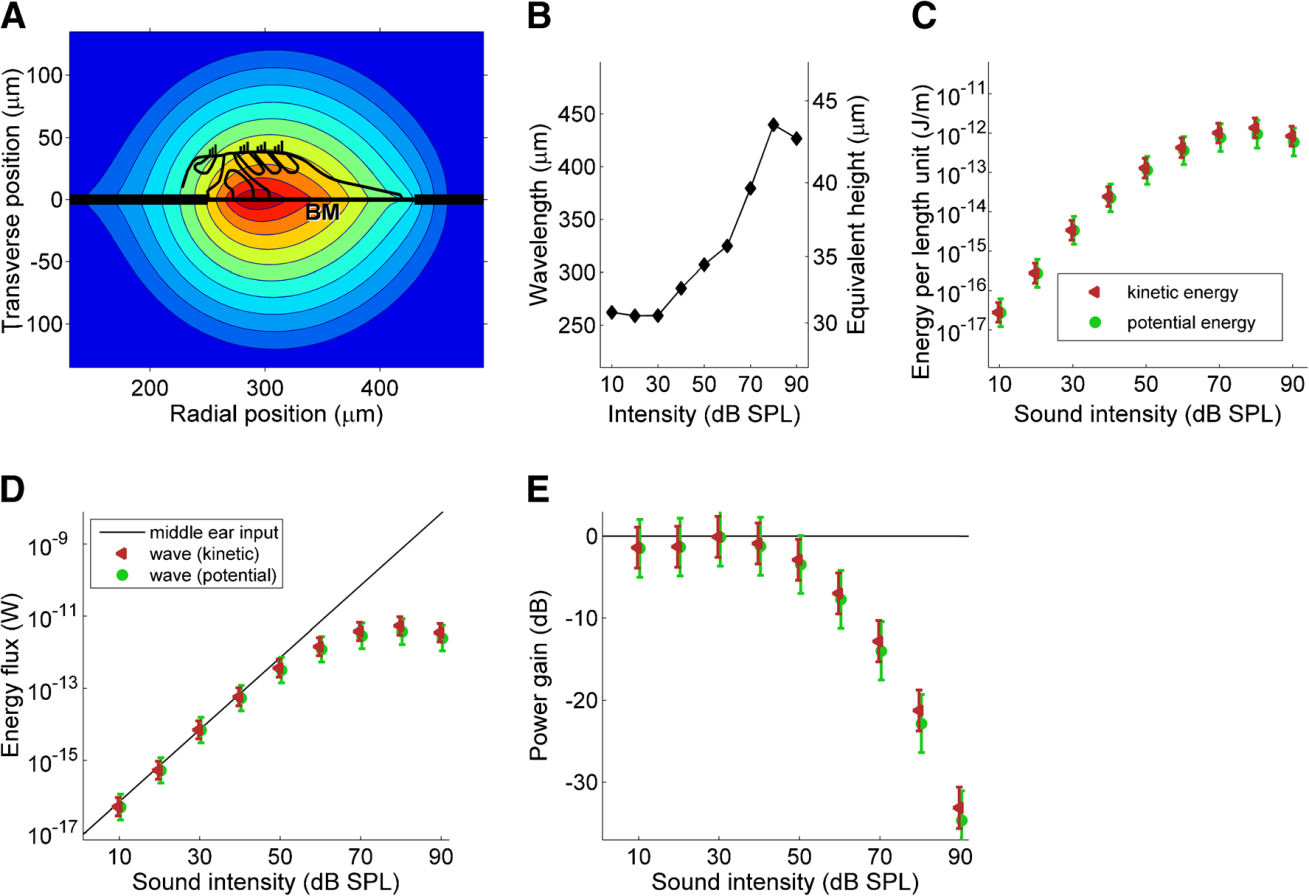
Net power gain in the traveling wave. **A** Cochlear cross section showing the spatial distribution of fluid velocity magnitude (16 kHz, 50-dB-SPL tone). Contour spacing 3 dB (twofold reductions in kinetic energy). Organ of Corti sketched for reference. **B** Intensity dependence of wavelength at 16 kHz. Right abscissa: equivalent thickness of fluid motion (see text). **C** Energy density estimates derived from BM data of Ren et al. (2011a). *Triangles*: kinetic energy per unit length, summed over the cross section, of the 16-kHz wave at peak location, with *error bars* based on across-animal variation of BM displacement. *Circles*: potential energy, with *error bars* combining across-animal variation of BM displacement with spread in BM stiffness data. **D** Energy flux of the 16-kHz traveling wave at the 16-kHz place of the gerbil cochlea, derived from the data in the previous panel, using the *same symbols* and *colors. Solid line*: middle ear power input. **E** Net power gain from middle ear to traveling wave peak, derived from the data in previous panel. *Error bars* in **D**, **E** derive from across-animal standard errors in Ren et al. (2011a).

Next, we estimated both the kinetic and potential energy densities in the cochlea. The equality of their time averages (Lighthill 1978) provides an important cross-check. In order to estimate the kinetic energy, three-dimensional motion of the fluid surrounding the BM was computed from known spatial profiles of BM motion (Ren et al. 2011a) using a fluid-dynamic analysis (see “Analysis of Fluid Motion” section in “Methods”). From the three-dimensional fluid motion, the cross-sectional distributions of velocity magnitude were determined (Fig. 4A). Fluid motion was found to decay exponentially with distance from BM, characterized by an “equivalent thickness” (Steele and Taber 1979b). Wavelength varied strongly with intensity (Ren et al. 2011a); equivalent thickness varied in proportion (Fig. 4B). Thus, fluid motion was confined to smaller cross sections for softer sounds. Kinetic energy distributions were computed for each intensity and spatially integrated.

Potential energy was obtained by combining BM stiffness data (Emadi et al. 2004) with data on BM displacement (Ren et al. 2011a). A point stiffness of 0.79 N/m at the 16-kHz place of the gerbil cochlea measured by Emadi et al. (2004) with a probe tip diameter of 25 to 50 μm amounts to a stiffness per unit length *s* = 2.1 × 10^4^ N/m^2^. Alternative estimates from the literature are addressed in “Discussion” section. The time average potential energy per unit length equals ¼ *sξ*_BM_^2^, where *ξ*_BM_ is the displacement amplitude at the peak of the wave, extracted from Figure 4A of Ren et al. (2011a). The potential and kinetic energy estimates matched well (Fig. 4C). Their slight (1.7 dB) divergence toward high intensities may be attributed to the fact that *in vitro* stiffness data, measured using a glass fiber, do not incorporate the effect of stimulus intensity, whereas the wavelength data of Figure 4B suggest that *in vivo* stiffness increases somewhat with intensity.

The energy flux of the wave at its peak was obtained by multiplying group velocity and energy density (Fig. 4D). These estimates were compared to the power input to the middle ear *P*_ME_ = Re(*p*^2^/*Z*_ME_), with *p* the RMS sound pressure near the eardrum and *Z*_ME_ the middle ear impedance. From Figure 11 of Ravicz et al. (1992), *Z*_ME_ at 16 kHz is real and equal to 6 × 10^7^ Pa s/m^3^. At 0 dB SPL, the RMS sound pressure is 20 μPa, yielding *P*_ME_ = 6.67 × 10^-18^ W at 0 dB SPL. Alternative estimates from the literature are addressed in “Discussion” section.

Up to 40 dB SPL, traveling wave power was slightly less than the acoustic middle ear input; at higher intensities, it fell behind. The net power gain from middle ear to the peaking wave (Fig. 4E) was never positive. Thus, we found no indication of a net power gain. Averaged over intensities ≤40 dB SPL, the gain was −1.0 ± 0.6 dB. Thus, 20 ± 11 % of the power is lost. With increasing intensity, the gain dropped to −34 dB. Thus, at 90 dB SPL, only 0.04 % of the energy entering the ear actually reached its characteristic place in the cochlea.

### Local Power Gain

The amplitude of the traveling wave is known to exhibit a local peak near its best place, particularly at low SPLs (e.g., Ren et al. 2011a). The rising flank of the peak necessarily exhibits a local amplitude gain. This, however, does not necessarily imply that there is also a local *power* gain. That the wave amplitude may well grow without injecting energy is illustrated by sea waves approaching the beach. Their growth is not caused by a coastal amplifier. The propagation speed depends on depth: when the wave enters shallower water, group velocity decreases (Lighthill 1978), causing an energy densification (“congestion”) that boosts the amplitude. Although this geometry of shoaling does not apply to the cochlea, the inverse relation between local group velocity and local wave amplitude is a general property of traveling waves (Whitham 1974), and there may exist in the cochlea other factors that affect group velocity. Amplitude peaking by wave deceleration occurs in passive resonant models (Lighthill 1981), but the idea is more general: it also applies to scenarios in which wave dispersion has nothing to do with resonance (Ranke 1950; Van der Heijden 2014).

**FIG. 5:**
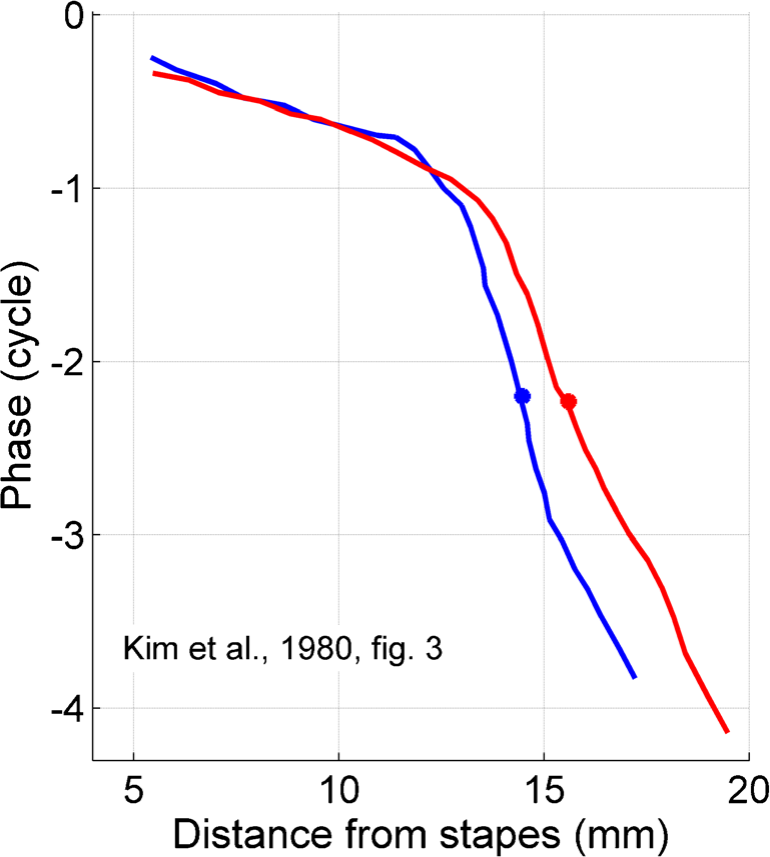
Deceleration of cochlear traveling waves shown by panoramic neural data. These data were extracted from Figure 3 of Kim et al. (1980a). The *curves* show the phase of many AN fibers of a single ear of the cat in response to the same tone pair of 2100 and 2700 Hz. The abscissa is the cochlear position derived from the BFs of the fibers. The distinct bend of the curves reflects the deceleration of the waves, which occurs over a narrow region just basal to the characteristic place of the tone marked by the *filled circles*.

Experimentally, wave deceleration in the cochlea is apparent from panoramic neural measurements, obtained by comparing the responses to the same stimulus across many single nerve fibers. These data show that the cochlear traveling wave slows down quite abruptly just prior to peaking (Kim et al. 1980a; Van der Heijden and Joris 2006; Palmer and Shackleton 2009; Temchin et al. 2012). An example of this abrupt deceleration is shown in Figure 5, which reproduces spatial phase profiles in a single cochlea from Kim et al. (1980a). The key question is: can the local amplitude growth be accounted for by the local deceleration of energy transport? If so, there is no obvious role for power amplification. We addressed this question by studying local amplitude gain and group velocity in finer detail.

**FIG. 6:**
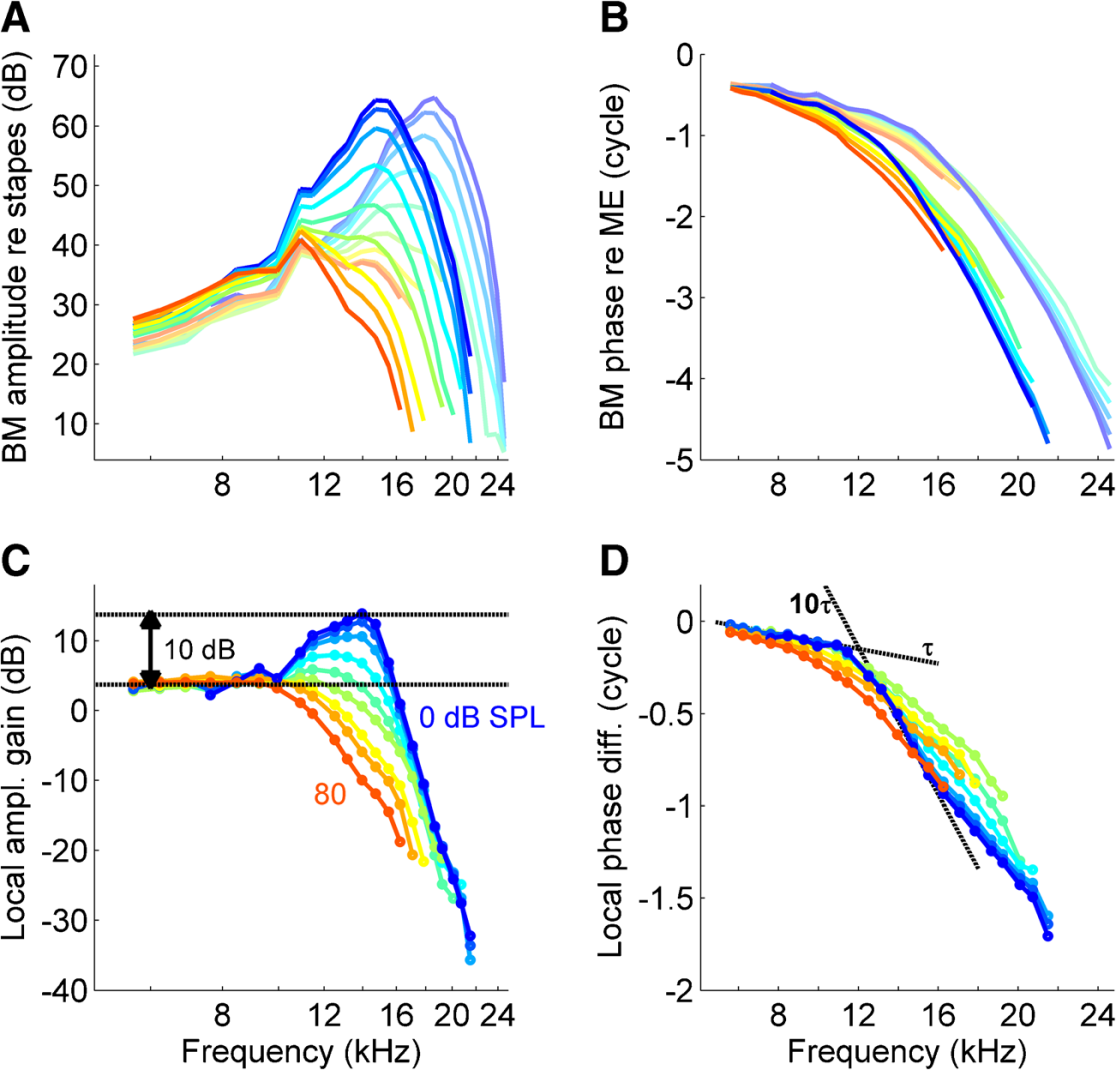
Local amplitude gain and the deceleration of energy transport. **A** BM amplitude normalized to stapes motion in response to tone complexes presented at 0 to 80 dB SPL per component in 10-dB steps. *Each curve* represents a single recording. The two sets of curves were obtained from two adjacent locations in the same cochlea (BF 15.0, 18.3 kHz), the *darker curves* corresponding to the more basal location. **B** Companion phase curves. **C** Local transfer functions obtained by plotting the local gain (i.e., amplitude ratio across the two locations) against frequency. *Lower black line* marks the low-frequency limit to which all the curves converge, independent of intensity. This linear limit serves as the reference for the excess gain of the intensity-dependent portions of the curves. *Upper black line* marks the largest (10-dB) excess gain occurring in this dataset. **D** Companion phase curves, showing the phase difference between the two locations. The sharp bend at low intensities signals the abrupt deceleration of energy transport just prior to the wave peaking. The *black lines* match the slopes of the 0-dB-SPL curves (*dark blue*) at both sides of the transition. Their slopes differ by a factor of 10, marking the tenfold deceleration of the 0-dB-SPL curve (animal RG12446).

We measured *in vivo* BM vibrations at two adjacent locations, this time using a wideband multitone stimulus presented at various SPLs (see “BM Recordings” section in “Methods”). Figure 6A shows two sets of magnitude curves, normalized to stapes motion. Stapes-to-BM amplitude gain decreased with increasing intensity, reflecting the compressive nature of the BM response. Compression was evident at the lowest intensities used (0 dB SPL per tone), indicating the high sensitivity of this cochlea. Figure 6B shows the companion phase curves. From these two sets of single-point data, we constructed the local transfer functions as introduced in Ren et al. (2011b) by plotting the amplitude ratio and phase difference between the two locations against frequency.

The local amplitude gain functions (Fig. 6C) quantify the wave peaking. The low-frequency portion was flat and invariant with intensity. In this linear, passive range, the amplitude ratio between adjacent locations is fixed by the BM stiffness gradient. We used this “passive amplitude ratio” of this linear range (*lower horizontal black line* in Fig. 6C) as the reference for the amplitude gain in the nonlinear, “active” range (see “Relation Between Wave Amplitude and Group Velocity” section in “Methods” for mathematical underpinning). At higher frequencies, local gain became strongly intensity dependent. This compressive range is commonly associated with amplification. Positive amplitude gains were observed only at low sound intensities (<40 dB SPL). The largest gain (∼10 dB above the low-frequency reference, marked by the *two-sided arrow* in Fig. 6C) occurred for the lowest intensity (0 dB SPL) just below local best frequency (BF). This maximum local gain captures the steepest part of the growing flank of waves on their way to peaking beyond the recording site.

The companion phase transfer functions (Fig. 6D) curve downward, independent of sound intensity. As the slopes correspond to group delay, this means that low-frequency energy travels faster than high-frequency energy. Considering the frequency-place map, this implies that the energy slows down as it travels, in agreement with the neural data reproduced in Figure 5. The deceleration occurs just prior (basal) to the wave’s peak region. Deceleration was abrupt at the low intensities (Fig. 6D, *blue curves*) and turned smoother with increasing intensity. For the softest sounds (0 dB SPL), a <20 % increment in frequency produced a tenfold reduction in group velocity (*black lines* in Fig. 6D). Since the best frequencies of the two recording locations differed by ∼20 %, the group velocity of waves near the transition frequency fell tenfold between them. This tenfold deceleration should cause a 10-dB increase in energy density, a prediction matched by the observed ∼10-dB amplitude gain (two-sided arrow in Fig. 6C). The quantitative match between phase bends and amplitude gain was a general finding in sensitive cochleae. The local amplitude gain was accounted for by the deceleration of the wave, and we found no indication of local power gain.

With increasing intensity, amplitude gain diminished and turned into loss (Fig. 6C). Notice that the transfer remained strongly compressive: the gain, even when negative, continued to decrease with intensity. Thus, at these high intensities, there was a local amplitude reduction at all frequencies in the nonlinear range. This shows at once that at these intensities, the active mechanism that creates the compression acts as a brake rather than an amplifier (see “Introduction” section). In order to estimate the local power loss at high intensities, one needs to combine the local amplitude ratio with the change in group velocity (just as was done for the 0-dB-SPL case above). The 80-dB-SPL local phase transfer at 14 kHz shows a sevenfold reduction in group velocity. In the absence of damping, this deceleration of energy transport would lead to an 8-dB local amplitude gain. The observed 14-dB amplitude reduction (a gain of minus 14 dB) therefore corresponds with a 22-dB power loss caused by local damping. Given the 0.6-cycle local phase difference, this corresponds to a power loss of 36 dB per wave cycle, indicating that the local wave propagation at 14 kHz, 80 dB SPL, was underdamped with a dimensionless damping coefficient *ζ* = 0.22 (see “Degree of Damping of Waves” section of “Methods”). Notice that 14 kHz is below the BF of the more apical of the two recording sites, so the 14-kHz wave has not reached its own characteristic place. Thus, a substantial amount of the energy of high-intensity sounds is absorbed in the region situated basal to their tonotopic place. The large size of the local losses and the place where they occur are consistent with our earlier observation (Fig. 4E) that only a minute fraction of the acoustic energy of intense sounds reaches the characteristic location. The bulk is dissipated just beforehand.

**FIG. 7:**
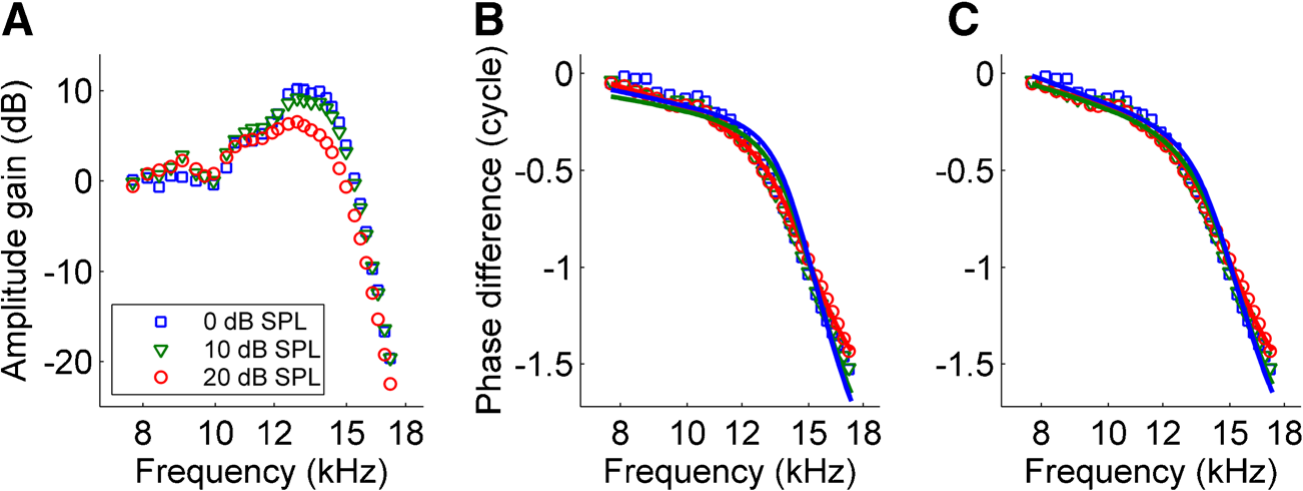
Predicting local phase from local amplitude gain, assuming zero power gain. **A** Low-intensity local amplitude gain obtained with fine frequency spacing. Intensity per component is indicated in the graph. **B** Companion local phase curves. *Symbols*: data. *Lines*: prediction computed from the local amplitude data under the assumption that there is no power gain, i.e., that the only source of the local amplitude gain is the deceleration of the wave (see text). The predictions accounted for 96.9 % of the variance in the phase data. **C** As in **B**, but now allowing for a 1.8-dB near miss for the two lowest intensities (see text), which eliminates the small systematic deviations observed in **B** (animal RG12436).

To further analyze the positive amplitude gain at low intensities, we performed recordings using finer frequency spacing. The local transfer functions (Fig. 7A, B) showed the same trends as observed in Figure 6: a positive local amplitude gain just below the lowest BF and a sharp phase bend. Notice the subtle effects of the 20-dB variation of stimulus intensity: a reduction of the amplitude gain and a slight smoothing and reduction of the phase bend. We derived the mathematical relation between local amplitude gain and phase transfer from the Ansatz that there is neither net energy injection nor net dissipation, causing the spatial gradient in group velocity to be the major determinant of amplitude variation. We used this mathematical relation to predict from the observed amplitude gain the associated phase transfer (see “Relation Between Local Amplitude Gain and Local Phase Difference” section in “Methods”). The predictions (Fig. 7B, *lines*) correctly predict the location and shape of the bend. For the lowest two intensities, calculated phase curves were slightly too steep. This near miss disappeared when allowing for a 1.8-dB underestimation of the gain for the 0- and 10-dB-SPL curves (Fig. 7C). The near miss suggests a small contribution of a secondary factor (in addition to reduction of group velocity), e.g., a simultaneous reduction in effective stiffness or a very slight (<2 dB) amplification after all (see “Discussion” section). Importantly, the calculations faithfully reproduced the observed correlation between amount of gain and sharpness of the phase bend, again emphasizing the intimate relation between amplitude growth and deceleration. Their quantitative match again suggests that the amplitude gain is not created by energy injection into the wave, but by the densification following the deceleration of energy transport by the wave.

## DISCUSSION

### Summary

We estimated the net power gain from middle ear to the peak of the traveling wave in two independent ways. The first method, based on kinetic energy, used the detailed spatial profile of BM motion (Ren et al. 2011a) to derive the motion of the surrounding fluid. In the second method, we estimated the potential energy from BM stiffness data (Emadi et al. 2004). These two independent energy estimates were combined with our own measurements of group velocity. The two methods yielded highly similar values for the energy flux, neither of which exceeded the middle ear power input at any intensity. At high intensities, there was a large (>30 dB) net loss. Thus, we did not find evidence for a net power gain at low intensities and clear evidence for a net power loss at high intensities.

In a second series of experiments, we performed two-point BM recordings and analyzed the local energy flux. We observed a steep deceleration of the wave that was sufficient to explain its peaking. We found no local power gain between adjacent points on the BM at any intensity. At high intensities, there was a strong local power loss (36 dB per traveled cycle just below BF).

### Uncertainties in the Estimates of Net Power Gain

The determination of net power gain from middle ear to the peaking wave necessarily involved combining diverse sources of published data. This introduces some uncertainty, especially when the sources disagree. A fortunate circumstance was the availability of all necessary data for the 16-kHz range of the gerbil: no extrapolation was needed.

Uncertainties in BM stiffness estimation are due to methodological challenges including the need to use post mortem preparations, anisotropy of the anatomical structures, and sensitivity to ion concentrations of the bath (Emadi et al. 2004). The stiffness values of Naidu and Mountain (1998) were systematically higher than those of Emadi et al. (2004) employed here. Underneath the OHCs, 2.5 mm from the base, their stiffness values exceed that of Emadi et al. by a factor of 3.4. Had we used the data of Naidu and Mountain (despite the methodological issues concerning those data raised by Emadi et al.), the estimate of energy flux based on the potential energy (*circles* in Fig. 4D) would be elevated by 5.3 dB, yielding a 4.3-dB net power gain from middle ear to BM at low intensities. But this would also introduce a ∼5-dB discrepancy between kinetic and potential energy estimates, because the former are independent of BM stiffness.

Middle ear losses present another source of uncertainty, equally affecting the estimates based on kinetic and potential energy. If, as suggested by de la Rochefoucauld et al. (2008), only 30 % of the power entering the middle ear is transferred to the cochlea, this would elevate the energy flux estimates by 5.2 dB, leading to a 4.2-dB value of the net power gain at low intensities. Figure 11 of de la Rochefoucauld et al. (2008) provides two alternative estimates of power input to the cochlea, based on previous work (Olson 1998; Dong and Olson 2006). At 16 kHz, 0 dB SPL, these cochlear power input estimates amount to 3.8 × 10^−18^ W and 1.2 × 10^−18^ W, respectively. Using these values (instead of the 6.7 × 10^−18^ W value used to compile Fig. 4E) would yield low-intensity, net power gain estimates of 1.4 and 6.5 dB, respectively. Notice that these alternative values were obtained using considerably more invasive techniques (e.g., drilling a hole in the bony wall near the stapes) than the data of Ravicz et al. (1992) that led to our minus 1-dB estimate of net power gain at low intensities. Perhaps more importantly, any uncertainties in acoustic power input are irrelevant in the measurement of *local* power gain between adjacent cochlear locations, which we found to be vanishing at low intensities and increasingly negative at higher intensities (Figs. 6 and 7). Obviously, there can be no net gain without local gain.

Considering the smallness of the potential corrections and the mutual consistency of our three independent estimates, it is difficult to reconcile our findings with even a modest (5 dB) amount of power amplification. In this respect, we fully confirm the conclusions of Allen and Fahey (1992). Our findings cast reasonable doubt on the existence of cochlear power amplification and invalidate the much larger (>20 dB) estimates of power gain found in previous studies as discussed below. In addition, our results show that the propagation of waves at high intensities is strongly dissipative. This is significant because the cochlea’s dynamic compression persists to the high-intensity range. Apparently, the active mechanism works as a variable attenuator (“brake”) rather than an amplifier at these intensities. Likewise, our findings invalidate claims of negative damping persisting to high intensities discussed below; if those claims were correct, *positive* local power gains should be observed.

### Previous Estimates of Power Gain and Negative Partition Impedance

Brass and Kemp (1993) used a similar scheme to deduce energy flux from BM recordings and found significant power amplification. The crucial difference between their study and ours is the assessment of group velocity. Because they worked with single-point recordings from Robles et al. (1986), they were unable to assess group velocity directly and had to revert to extrapolations based on tonotopy. Moreover, the limited S/N ratio of the data (obtained with a Mössbauer source) necessitated considerable numerical smoothing of data in order to estimate group velocity. The resulting estimated dependence of group velocity on position (Fig. 2B of Brass and Kemp 1993) is very shallow. In contrast, we determined group velocity by a direct comparison of magnitude fluctuations between adjacent locations (Fig. 3), did not apply any smoothing, and found a steep transition in group velocity when varying frequency (Figs. 6 and 7). The steepness is a key observation of the current study, and the inferred abrupt deceleration of energy transport is a key step in reaching the conclusion that there is no local power amplification.

Several studies have used “inverse methods” to estimate BM impedance or the amount of power gain from BM data (Zweig 1991). While the estimates of power amplification thus derived are highly variable, some of these studies cannot be reconciled with our current findings: negative damping up to high intensities (e.g., 80–90 dB SPL in octave-wide bands, de Boer and Nuttall 2000) and power gain estimates of up to 17.7 dB (de Boer and Nuttall 2001). Unlike Brass and Kemp (1993) and the current study, the inverse method uses an explicit fluid dynamical model in which the cochlear partition is treated as point impedance *z*_BM_(*x*,*ω*), a quantity that depends on frequency *ω* and place *x*, but not directly on wavelength. Estimates of *z*_BM_(*x*,*ω*) are obtained by fitting the model to (extrapolated) single-point BM data. Claims of amplification follow from any negative real part of *z*_BM_ thus obtained (“negative damping”).

The central assumption of the inverse method is the adequacy of point impedance to describe how the cochlear partition interacts with the fluid. This assumption is not self-evident (see also Brass and Kemp 1993), as it ignores the finite dimensions and internal structure of the organ of Corti. That simplification would be justified if the organ were small compared to the typical scale of the wave (the distance over which relative motion becomes comparable to absolute motion), but this is not the case for near-BF waves. In the transverse direction (“depth”), the motion of fluid participating in a wave falls off exponentially, having a penetration depth of ∼*λ*/2π (Lighthill 1978; Steele and Taber 1979a). Near BF, the wavelength is ∼250 μm (Fig. 4C), so the penetration depth equals ∼40 μm. Because the height of the organ of Corti in the 16-kHz region well exceeds 50 μm (Edge et al. 1998), the amplitude of fluid motion varies at least threefold over its height. Thus, for these shorter wavelengths, the constituents of the partition need not move uniformly. Any nonuniform motion involves periodic internal deformations that are unlikely to be captured by a point impedance description. Only for much longer (lower-frequency) waves is the motion virtually uniform.

Intriguingly, the transition between these regimes, which takes place when the wavelength drops below a millimeter, is situated just basal to the peak—exactly in the region of alleged amplification. As long as one holds on to a point impedance description, it seems inevitable to invoke some degree of negative damping to be able to explain experimental data (de Boer 1995; Olson 2001). But if one relaxes the assumption of point impedance, negative damping may well become unnecessary. In terms of inverse methods, this amounts to allowing *z*_BM_ to depend not only on position and frequency, but also explicitly on wavelength: *z*_BM_ = *z*_BM_(*x*,*ω*,*λ*). This generalized notion of partition impedance paves the way to explanations of amplitude growth that are more in line with the main experimental finding of this study: the abrupt deceleration of energy transport. An explicit implementation of this approach is the hydrodynamic waveguide model of Van der Heijden (2014). This passive, linear model has two coupled elastic beams (“membranes”). Its traveling waves exhibit *mode shape swapping*, a rapid change of the relative beam motion in the course of propagation. The transition reduces both the group velocity and the effective stiffness, and both reductions contribute to a local amplitude boost (the latter contribution, from the stiffness reduction, may explain the near miss mentioned in connection with Fig. 7). As discussed in that study, mode shape swapping mimics the behavior of active models: over a narrow spatial region, the wave amplitude is boosted. This boost, however, is not created by motile activity, but by the rapid transfer of power from a nondispersive and stiff vibration mode into a highly dispersive and compliant one. Yet, an observer watching one beam, but unaware of the other, would be tempted to attribute the sudden boost to a local power source. Being unaware of the other beam, the observer misses the spatially distributed (nonpoint-like) character of the impedance of the beam pair.

At first glance, the questioning of point impedance may appear to undermine our own analysis of net power gain (Fig. 2), because that does not take into account the finite size of the partition, either. Note, however, that the analysis deals with the vibration right at the peak of the traveling wave amplitude, whereas the region of alleged amplification is just basal to the peak. What makes the observer of the previous paragraph believe in amplification is not the magnitude of the peak *per se*, but the steep amplitude growth leading to the peak. Viewed from that perspective, the problem is not an unduly large peak magnitude, but the unexpected smallness of the wave magnitude just basal to the peak. The steep growth itself is more puzzling than its end product, the peak magnitude. While the acoustic power entering the cochlea is sufficient to create the peak magnitude, on its way there, the power appears to be somehow contained, i.e., prevented from generating the magnitude of local motion that it could afford. The sharp peak is then created by rapidly unleashing the power. The waveguide model of Van der Heijden (2014) demonstrates a possible physical mechanism of this unleashing, namely, a transition of vibration mode. At the peak (beyond the transition), the waves in that model are of the three-dimensional, “fanning” type (Steele and Taber 1979b), for which the cochlear partition is well approximated by a point impedance. It is in the transition region just basal to the peak that the point impedance description breaks down.

### Cochlear Attenuation

Although it cannot be entirely excluded that the lack of power gain stems from a near-perfect balance between power amplification and ordinary dissipation, the most parsimonious interpretation of our findings is that there is no amplifier, that cochlear sensitivity is not realized by amplifying acoustic energy, but by spatially focusing it, and that dynamic compression is realized by locally adjusting the amount of dissipation to sound intensity. We end by briefly exploring the physiological and functional implications of such an interpretation. While not solving all known problems in cochlear mechanics, it offers alternative solutions to some and sheds new light on others.

The change of perspective from a saturating amplifier to a variable attenuator has remarkably little impact on the character of cochlear responses: both schemes predict compression, two-tone suppression, and distortion products (Van der Heijden 2005). But the underlying physiological mechanisms differ greatly. Mechanical amplification is physiologically demanding, as it involves positive feedback which is phase locked to high-frequency waveforms. Its problematic aspects include uncontrollable instabilities, severe shunting of the input to motile elements due to low-pass filtering, and lack of a clearly identified mechanism to couple high-frequency motile output to BM vibration (Ashmore et al. 2010). Cochlear attenuation is less demanding. It still requires an active process that regulates dissipation, but its feedback is negative, minimizing (though not necessarily eliminating) stability problems. Moreover, it involves a straightforward friction control, comparable to the brakes of a car. Just like operating a brake requires no synchronization to the wheel rotation, a cochlear attenuator can do without phase locking to high-frequency stimuli. This eliminates the remaining physiological problems listed above. OHCs, whose length can change with their membrane potential, play a crucial role in cochlear compression, and damaging them immediately reduces sensitivity. The exact mechanism by which OHCs control the vibrations is unknown, but if we assume that OHC shortening acts to increase local friction, this explains both the dynamic compression (through sound-induced depolarization) and reduced sensitivity with trauma (through loss of turgor). Neither phenomenon specifically favors amplification over attenuation.

The analysis of abrupt phase bends (Fig. 6) showed that the peaking of cochlear waves is quantitatively explained by the focusing of wave energy through selective deceleration. Sharp phase bends are also apparent in panoramic studies that pool phase data from large numbers of auditory nerve fibers in cat (Kim et al. 1980a; Van der Heijden and Joris 2006), guinea pig (Palmer and Shackleton 2009), and chinchilla (Temchin et al. 2012). Thus, abrupt wave deceleration is a general cochlear phenomenon. The smoothing of deceleration at high intensities (discussed in connection with Figs. 6 and 7) is also a general finding in mammals (Versteegh and Van der Heijden 2013). Smoothing has an interesting consequence: the basalward extension of the region over which the energy transport is slowed down. Because slowing down the energy transport enhances the spatial rate of dissipation, this suggests that the “premature deceleration” contributes to compressing the dynamic range, in addition to the direct control of damping. This additional mechanism would be especially useful at the highest intensities, when large local power losses are required to curb the spatial overlap of spectral components.

The insight that the cochlea exerts a form of mechanical sensitivity control predates models based on cochlear amplification (Rose et al. 1971; Kim et al. 1973). The functional necessity of a stage of dynamic range compression prior to transduction was recognized and analyzed by Allen (1979), who proposed a framework of “nonlinear damping [that] acts as a mechanical automatic gain control.” The results of the present study support this view in which damping is an asset rather than a drawback. The detection of faint tones, however useful, is not the major task of most ears. In the daily life of many species, spectral analysis and dealing with noisy environments are much more common tasks, which impose nontrivial challenges of an entirely different nature. Because the cochlea is a waveguide, its function as a spectral analyzer requires that it absorb all acoustic power entering it. Any reflected component will interfere with the processing of higher frequencies; any component traveling beyond its proper region will interfere with lower frequencies. If the cochlea is to resolve individual components whose intensities differ by several orders of magnitude, the absorption must be both well placed and rigorous. Viewed in this light, dissipation is an indispensable tool rather than something that must be “overcome by amplification,” and it is to be expected that the mammalian cochlea has developed a fine control over the amount of local dissipation.

## References

[CR1] Allen JB, Hoke B, de Boer E (1979). Cochlear models—1978. Models of the auditory system.

[CR2] Allen JB, Fahey PF (1992). Using acoustic distortion products to measure the cochlear amplifier gain on the basilar membrane. J Acoust Soc Am.

[CR3] Ashmore J (2008). Cochlear outer hair cell motility. Physiol Rev.

[CR4] Ashmore J, Avan P, Brownell WE (2010). The remarkable cochlear amplifier. Hear Res.

[CR5] Brass D, Kemp DT (1993). Analyses of Mössbauer mechanical measurements indicate that the cochlea is mechanically active. J Acoust Soc Am.

[CR6] Cody AR, Russell IJ (1987). The responses of hair cells in the basal turn of the guinea-pig cochlea to tones. J Physiol.

[CR7] Cooper NP (1998). Harmonic distortion on the basilar membrane in the basal turn of the guinea-pig cochlea. J Physiol.

[CR8] Cooper NP (1999). Vibration of beads placed on the basilar membrane in the basal turn of the cochlea. J Acoust Soc Am.

[CR9] Cooper NP (2000). Radial variation in the vibrations of the cochlear partition. Recent developments in auditory mechanics.

[CR10] De Boer E (1995). The “inverse problem” solved for a three-dimensional model of the cochlea. II Application to experimental data. J Acoust Soc Am.

[CR11] De Boer E, Nuttall AL (2000). The mechanical waveform of the basilar membrane. III. Intensity effects. J Acoust Soc Am.

[CR12] De Boer E, Nuttall AF, Breebaart D, Houtsma AJ, Kohlrausch A (2001). Power gain of the cochlear amplifier. Physiological and psychological bases of auditory function.

[CR13] De Boer E, Nuttall AL, Hu N (2005). The Allen-Fahey experiment extended. J Acoust Soc Am.

[CR14] De la Rochefoucauld O, Decraemer WF, Khanna SM, Olson ES (2008). Simultaneous measurements of ossicular velocity and intracochlear pressure leading to the cochlear input impedance in gerbil. J Assoc Res Otolaryngol.

[CR15] Dong W, Olson ES (2006). Middle ear forward and reverse transmission in gerbil. J Neurophysiol.

[CR16] Dong W, Olson ES (2009). *In vivo* impedance of the gerbil cochlear partition at auditory frequencies. Biophys J.

[CR17] Doyle JC, Francis BA, Tannenbaum AR (1992). Feedback control theory.

[CR18] Duke TAJ, Jülicher F, Manley G, Fay R, Popper A (2008). Critical oscillators as active elements in hearing. Active processes and otoacoustic emissions in hearing.

[CR19] Edge RM, Evans BN, Pearce M (1998). Morphology of the unfixed cochlea. Hear Res.

[CR20] Emadi G, Richter CP, Dallos P (2004). Stiffness of the gerbil basilar membrane: radial and longitudinal variations. J Neurophysiol.

[CR21] Evans EF, Harrison RV (1976). Proceedings: correlation between cochlear outer hair cell damage and deterioration of cochlear nerve tuning properties in the guinea-pig. J Physiol.

[CR22] Frank G, Hemmert W, Gummer AW (1999). Limiting dynamics of high-frequency electromechanical transduction of outer hair cells. Proc Natl Acad Sci U S A.

[CR23] Hudspeth AJ (2013). The inner ear. Principles of neural science.

[CR24] Kemp DT (1979) Evidence of mechanical nonlinearity and frequency selective wave amplification in the cochlea. Arch Otol Rhinol Laryn 224(1–2):37–45 10.1007/BF00455222 485948

[CR25] Kennedy HJ, Evans MG, Crawford AC, Fettiplace R (2006). Depolarization of cochlear outer hair cells evokes active hair bundle motion by two mechanisms. J Neurosci.

[CR26] Kim DO, Molnar CE, Pfeiffer RR (1973). A system of nonlinear differential equations modeling basilar-membrane motion. J Acoust Soc Am.

[CR27] Kim DO, Molnar CE, Matthews JW (1980). Cochlear mechanics: nonlinear behavior in two-tone responses as reflected in cochlear-nerve-fiber responses and in ear-canal sound pressure. J Acoust Soc Am.

[CR28] Kim DO, Neely ST, Molnar CE, Matthews JW (1980b) An active cochlear model with negative damping in the partition: comparison with Rhode’s ante- and post-mortem observation. In: van den Brink G, Bilsen FA (eds) Psychological, physiological and behaviour studies in hearing. pp 7–14

[CR29] Lighthill MJ (1965). Group velocity. IMA J Appl Math.

[CR30] Lighthill J (1978). Waves in fluids.

[CR31] Lighthill J (1981). Energy flow in the cochlea. J Fluid Mech.

[CR32] Lim DJ (1986). Functional structure of the organ of Corti: a review. Hear Res.

[CR33] Martin GK, Lonsbyry-Martin BL, Probst R, Coats AC (1988). Spontaneous otoacoustic emissions in a nonhuman primate. I. Basic features and relations to other emissions. Hear Res.

[CR34] Meenderink SWF, van der Heijden M (2011). Distortion product otoacoustic emissions evoked by tone complexes. J Assoc Res Otolaryngol.

[CR35] Naidu RC, Mountain DC (1998). Measurements of the stiffness map challenge a basic tenet of cochlear theories. Hear Res.

[CR36] Neely ST (1985). Mathematical modeling of cochlear mechanics. J Acoust Soc Am.

[CR37] Olson ES (1998). Observing middle and inner ear mechanics with novel intracochlear pressure sensors. J Acoust Soc Am.

[CR38] Olson E (2001). Intracochlear pressure measurements related to cochlear tuning. J Acoust Soc Am.

[CR39] Palmer AR, Shackleton TM (2009). Variation in the phase of response to low-frequency pure tones in the guinea pig auditory nerve as functions of stimulus level and frequency. J Assoc Res Otolaryngol.

[CR40] Plassmann W, Peetz W, Schmidt M (1987). The cochlea in gerbilline rodents. Brain Behav Evol.

[CR41] Ranke OF (1950). Theory of the cochlea: a contribution to the hydrodynamics of the cochlea. J Acoust Soc Am.

[CR42] Ravicz ME, Rosowski JJ, Voigt HF (1992). Sound-power collection by the auditory periphery of the Mongolian gerbil Meriones unguiculatus. I: Middle-ear input impedance. J Acoust Soc Am.

[CR43] Ren T, Gillespie PG (2007). A mechanism for active hearing. Curr Opin Neurobiol.

[CR44] Ren T, He W, Gillespie PG (2011). Measurement of cochlear power gain in the sensitive gerbil ear. Nat Commun.

[CR45] Ren T, He W, Porsov E (2011). Localization of the cochlear amplifier in living sensitive ears. PLoS ONE.

[CR46] Rhode WS (1978). Some observations on cochlear mechanics. J Acoust Soc Am.

[CR47] Rhode WS (2007). Basilar membrane mechanics in the 6–9 kHz region of sensitive chinchilla cochleae. J Acoust Soc Am.

[CR48] Rhode WS, Recio A (2000). Study of mechanical motions in the basal region of the chinchilla cochlea. J Acoust Soc Am.

[CR49] Robles L, Ruggero MA (2001). Mechanics of the mammalian cochlea. Physiol Rev.

[CR50] Robles L, Ruggero MA, Rich NC (1986). Basilar-membrane mechanics at the base of the chinchilla cochlea. I. Input–output functions, tuning curves, and response phases. J Acoust Soc Am.

[CR51] Rose JE, Hind JE, Anderson DJ, Brugge JF (1971). Some effects of stimulus intensity on response of auditory nerve fibers in the squirrel monkey. J Neurophysiol.

[CR52] Shera CA (2003). Mammalian spontaneous otoacoustic emissions are amplitude-stabilized cochlear standing waves. J Acoust Soc Am.

[CR53] Shera CA, Gummer AW, Dalhoff E, Nowotny M, Scherer MP (2003). Wave interference in the generation of reflection- and distortion-source emissions. Biophysics of the cochlea: from molecules to models.

[CR54] Shera CA (2007) Laser amplification with a twist: traveling-wave propagation and gain functions from throughout the cochlea 10.1121/1.2783205 18189566

[CR55] Steele CR, Taber LA (1979). Comparison of WKB and finite difference calculations for a two-dimensional cochlear model. J Acoust Soc Am.

[CR56] Steele CR, Taber LA (1979). Comparison of WKB calculations and experimental results for three-dimensional cochlear models. J Acoust Soc Am.

[CR57] Talmadge CL, Tubis A, Wit HP, Long GR (1991). Are spontaneous otoacoustic emissions generated by self-sustained cochlear oscillators?. J Acoust Soc Am.

[CR58] Temchin AN, Recio-Spinoso A, Cai H, Ruggero MA (2012). Traveling waves on the organ of corti of the chinchilla cochlea: spatial trajectories of inner hair cell depolarization inferred from responses of auditory-nerve fibers. J Neurosci.

[CR59] Van der Heijden M (2005). Cochlear gain control. J Acoust Soc Am.

[CR60] Van der Heijden M (2014). Frequency selectivity without resonance in a fluid waveguide. Proc Natl Acad Sci U S A.

[CR61] Van der Heijden M, Joris PX (2006). Panoramic measurements of the apex of the cochlea. J Neurosci.

[CR62] Versteegh CPC, van der Heijden M (2012). Basilar membrane responses to tones and tone complexes: nonlinear effects of stimulus intensity. J Assoc Res Otolaryngol.

[CR63] Versteegh CPC, van der Heijden M (2013). The spatial buildup of compression and suppression in the mammalian cochlea. J Assoc Res Otolaryngol.

[CR64] Whitham GB (1974). Linear and nonlinear waves.

[CR65] Wier CC, Norton SJ, Kincaid GE (1984). Spontaneous narrow-band otoacoustic signals emitted by human ears: a replication. J Acoust Soc Am.

[CR66] Zweig G (1976). Basilar membrane motion. Cold Spring Harb Symp Quant Biol.

[CR67] Zweig G (1991). Finding the impedance of the organ of Corti. J Acoust Soc Am.

